# A Safe Natural Alternative to Phenylthiourea: Ethyl Acetate Extract of *Alchemilla vulgaris* for Zebrafish Embryo Depigmentation

**DOI:** 10.3390/ph19050714

**Published:** 2026-04-30

**Authors:** Muhammad Farooq Khan, Mohammad Ahmad Wadaan

**Affiliations:** Department of Zoology, College of Science, King Saud University, P.O. Box 2455, Riyadh 11451, Saudi Arabia; wadaan@ksu.edu.sa

**Keywords:** *Alchemilla vulgaris*, *Danio rerio*, kojic acid, melanin, phenyl thiourea, pigmentation

## Abstract

**Background**: Zebrafish (*Danio rerio*) embryos are transparent in early stages of embryonic development; however, pigment formation at later stages hinders internal organ visualization during imaging. Chemicals such as 1-phenyl-2-thiourea (PTU) and kojic acid, used to block pigmentation, pose significant toxicity risks to human health. Therefore, effective and risk-free depigmentation agents are needed. This study investigates the efficacy of *Alchemilla vulgaris* (Lady’s mantle) as a safe, natural alternative for zebrafish embryo depigmentation. **Methods**: *A. vulgaris* was extracted using four solvents of varying polarities and evaluated for depigmentation efficacy and toxicity. Gas chromatography–mass spectrometry (GC–MS) was used to identify major constituents of the extract. **Results**: Ethyl acetate extract was more effective at removing pigments than all other extracts, and exhibited the lowest toxicity compared to PTU and kojic acid. Ethyl acetate extract of *A. vulgaris* remained effective even when administered 48 h post fertilization (post-pigmentation), making it suitable for long-term experiments requiring optical clarity. GC-MS revealed that this extract was rich in linoleic acid, various fatty acid esters, and phenolics, which likely contributed to its depigmentation activity. **Conclusions**: Based on these findings, we propose that ethyl acetate extract of *A. vulgaris* is a safer, natural alternative to PTU and kojic acid for depigmenting zebrafish embryos, particularly in long-term imaging experiments. The extract exhibits high efficacy at low concentrations, accompanied by a favorable toxicity profile, demonstrating potential as a depigmentation agent during early zebrafish development. However, further studies are needed to elucidate its mechanism of action.

## 1. Introduction

The natural transparency of zebrafish embryos enables researchers to directly observe organ development in real time. This includes cell movement during gastrulation and the formation of the nervous system, heart, and other organs. It also facilitates the study of tumor progression and metastasis in cancer, as well as the effects of genetic mutations and drugs on internal structures. However, zebrafish embryos remain transparent only during the early stages (0–24 hpf). Thereafter, melanophores develop and produce melanin, resulting in dark pigmentation that diminishes optical clarity and hinders the observation of internal structures, including neural activity and vasculature. In addition to visibility, inhibiting pigmentation is essential for imaging techniques such as confocal, light, and fluorescence microscopy, as even tiny pigments can scatter light and substantially compromise image quality. Hence, for post-melanin observation, researchers treat embryos with lower concentrations of 1-phenyl-2-thiourea (PTU). This compound blocks tyrosinase, an enzyme necessary for melanin production [1], resulting in embryos remaining almost transparent during the later stages of development. However, the toxic effects of PTU extend beyond zebrafish embryos and may pose a serious threat to human health, particularly to researchers handling it. PTU has been reported to increase the risk of developmental defects in zebrafish when administered during the early stages (0–24 hpf) of embryonic development without enhancing optical clarity [2]. It induces metabolic abnormalities that aggravate the hepatotoxicity of bavachalcone [3] and causes a reduction in eye size in larval fish starting three days post fertilization [4]. PTU acts as an endocrine disruptor in zebrafish by inhibiting thyroid peroxidase (TPO), resulting in hypothyroid-like phenotypes including decreased thyroid hormone synthesis, smaller or malformed thyroid follicles, impaired growth, and craniofacial defects, which may pose risks to pregnant individuals [4,5]. Moreover, a recent study reported that PTU may cause neurological interference [6]. Additionally, PTU exposure in humans, either through ingestion or dermal absorption, may cause pleural effusion, hypothermia, difficulty in breathing, cyanosis, pulmonary edema, hepatotoxicity, and hyperglycemia [2,3,7]. Kojic acid is a carcinogenic compound that has also been used as a depigmentation agent in zebrafish research [8]. Therefore, its use in skin treatments is strictly regulated [9]. Moreover, its use is limited due to poor skin penetration and ineffectiveness in vivo [10].

These limitations highlight the need for alternative depigmentation agents that are effective at low concentrations, pose minimal health risks, and are cost-effective. While some plant extracts have been tested for depigmentation in zebrafish, crude extracts or isolated compounds are often effective only at higher concentrations that cause toxicity [11]. During routine screening of herbs and medicinal plants, we observed that the ethyl acetate extract of *Alchemilla vulgaris* (lady’s mantle and locally called lions’ foot (رجل الاسد)) inhibited pigment formation in zebrafish embryos at very low concentrations. Previous studies report that this plant is used in cosmetics for skin whitening and is considered safe for long-term use [12,13].

This study investigates whether *A. vulgaris* extracts provide a safer alternative for depigmentation in zebrafish embryos, considering the known toxicity of PTU and kojic acid. Although *A. vulgaris* is commonly used in dermatology, its safety profile, particularly in sensitive models such as zebrafish embryos, remains unexplored. Moreover, published studies are further limited by a narrow focus on the use of a single solvent (mostly alcoholic solvents); thus, a systematic characterization of the phytochemical profiles and comparative safety of *A. vulgaris* across diverse solvent systems is currently unknown.

We performed comparative analysis of depigmenting activity and developmental toxicity of *A. vulgaris* extracts prepared using four different solvents against PTU and kojic acid in zebrafish embryos, accompanied by phytochemical profiling to characterize active components.

## 2. Results

### 2.1. Determination of In Vivo Development Toxicity of A. valgaris Extracts in Zebrafish Embryos

The LC_50_ values for each solvent extract were equal to or more than 1 mg/mL after 12 h of treatment at 24 hpf (Table 1). However, CHCl3 and methanol extracts exhibited toxicity after 48 h of treatment with LC_50_ values of 127.18 ± 1.05 and 62.5 ± 1.75 µg/mL, respectively. Moreover, all treated embryos (n = 60) died after 96 h of treatment in CHCl_3_ extracts. CHCl_3_ extracts also induced significant teratogenic effects (developmental delay, delayed hatching, and pericardial edema) in treated embryos (*p*-value < 0.05). Zebrafish embryos treated with n-hexane (nHex) and ethyl acetate extracts of *A. vulgaris* exhibited minimal toxicity. PTU at a concentration of ≤30 µg/mL did not induce toxicity or teratogenic effects in zebrafish embryos, but was toxic at 100 µg/mL. Kojic acid induced toxicity in zebrafish embryos with LC_50_ values of 217.18 ± 1.67, and exposure at a concentration of 200 µg/mL (effective depigmentation dose) caused developmental delay and cardiac edema.

### 2.2. Ethyl Acetate Extract of A. valgaris as Depigmentation Agent in Zebrafish

**Figure 1:**
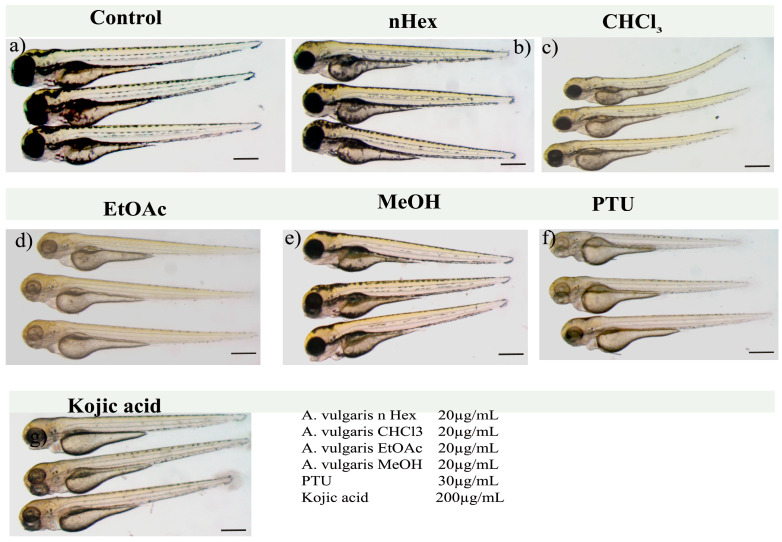
***A valgaris* extracts blocked the melanin formation in zebrafish embryos.** Representative micrograph of zebrafish embryos, mock control 0.5% *v*/*v* methanol (**a**), treated with extracts of *A. valgaris*, nHex (**b**), CHCl3 (**c**), ethyl acetate (**d**), methanol (**e**), PTU (**f**) and kojic acid (**g**). Significant reduction in pigment formation in zebrafish embryos was observed with the ethyl acetate extract of *A. valgaris* without any noticeable teratogenicity.

**Figure 2:**
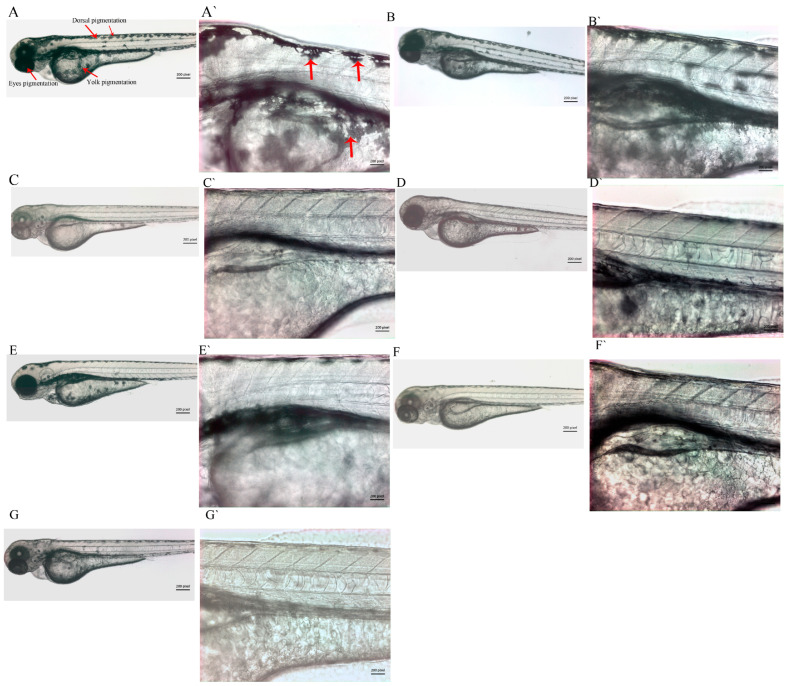
**Magnified (5× 20×) representative images of zebrafish embryos—mock control and treated with various extracts of *A. vulgaris*, PTU and kojic acid.** Control (**A**,**A`**), nHex (**B**,**B`**), ethyl acetate (**C**,**C`**), Chloroform (**D**,**D`**), methanol (**E**,**E`**), PTU (**F**,**F`**) and kojic acid (**G**,**G`**). The pigmentation is shown by the red arrows.

Zebrafish embryos were treated with four solvent extracts of *A. vulgaris*, and their depigmentation efficacy was compared with that of commonly used depigmentation chemicals, such as phenyl thiourea and kojic acid. The comparison criterion was the minimum chemical concentration required to stop melanin production without inducing severe toxicity or teratogenic effects in zebrafish embryos. CHCl_3_ and ethyl acetate extracts of *A. vulgaris* reduced melanin production in the treated embryos, whereas the nHex and methanol extracts did not (Figure 1 and Figure 2 higher magnification). The serial dilution experiments showed that 10 µg/mL of ethyl acetate extract of *A. vulgaris* was sufficient to inhibit melanin production significantly in zebrafish embryos, whereas the CHCl_3_ treatment required 20 µg/mL to produce a similar effect. PTU was used at the recommended standard concentration of 30 µg/mL (0.003%). However, 200 µg/mL of kojic acid was needed to effectively block melanin production in zebrafish embryos. The zebrafish embryos treated with *A. vulgaris* CHCl_3_ extract (20 µg/mL) also exhibited a significant reduction in pigments. However, a teratogenic effect (curved tail) was observed in 30% of the embryos. On the other hand, n-Hexane and methanol extracts were not active and did not block pigment formation in zebrafish embryos even at concentrations 10 times higher than those of ethyl acetate and chloroform extracts.

**Figure 3:**
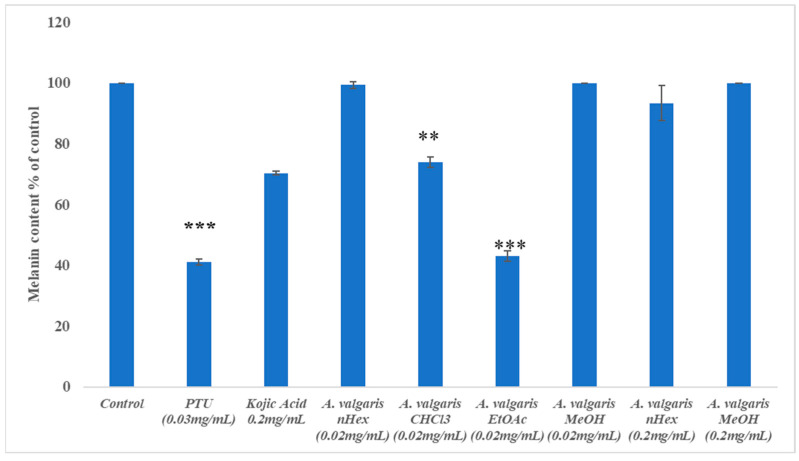
**Melanin inhibitory effect of *A. valgaris* extracts in zebrafish embryos.** Data represent mean ± SD of three independent experiments, each using pools of 10 embryos (n = 3 biological replicates). Statistical analysis was performed using one-way ANOVA with Dunnett’s post hoc test for comparisons against the control. Values are expressed as % of control. Error bars indicate standard deviation. *** *p* < 0.001 for EtOAc and PTU vs. control; ** *p* < 0.01 for CHCl_3_ vs. control.

Melanin content in zebrafish embryos treated with *A. vulgaris* extracts, PTU, and kojic acid was also measured by spectrophotometry (Figure 3). Zebrafish embryos treated with 20 µg/mL of ethyl acetate extract of *A. vulgaris* exhibited a 60% reduction in melanin content, comparable to PTU at 30 µg/mL, whereas the kojic acid at 200 µg/mL reduced melanin by only 30%. A 30% reduction in melanin content was observed with the CHCl_3_ extract of *A. vulgaris*. In contrast, no reduction in melanin content was observed with the methanol and nHex extracts of *A. vulgaris*, even when zebrafish embryos were treated at nearly 10 times the effective concentration (Figure 3).

**Figure 4:**
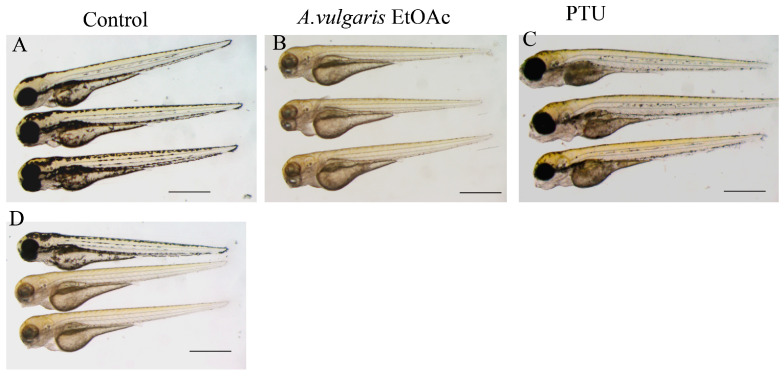
**Post-melanin synthesis reduction efficacy of *A. valgaris*.** Zebrafish larvae were treated with 20 µg/mL ethyl acetate extract or 30 µg/mL PTU at 48 hpf. Pigment formation was assessed at 72 hpf. The ethyl acetate extract-treated larvae showed significantly less pigment than PTU-treated larvae. (**A**) Control mock treated larvae at 72 hpf, showing dorsal and eye pigmentation; (**B**) zebrafish embryos treated with ethyl acetate extract of *A. Vulgaris*, showing reduced pigmentation; (**C**) zebrafish embryos treated with 30 µg/mL of PTU; (**D**) the control and ethyl acetate extract-treated embryos pictured at the same panel to keep the same light exposure conditions. Scale bar = 500 µm.

A notable characteristic of the ethyl acetate extract is its ability to reduce pigment levels after melanin synthesis. Zebrafish larvae treated with 20 µg/mL ethyl acetate extract at 48 hpf exhibited less pigment at 72 hpf compared with those treated with 30 µg/mL PTU (Figure 4). Quantification confirmed a 45% reduction in melanin content in the ethyl acetate-treated group compared to 30% in the PTU-treated group (*p* < 0.05).

Many green plants affecting melanogenesis contain substances of a photodynamic and phototoxic activity [14,15]. To determine whether the observed depigmentation resulted from biological activity rather than photobleaching, batches of embryos were treated with ethyl acetate extract and incubated at 28 °C under either light (14 h light, ~2000 lux) or complete darkness (14 h). Depigmentation was consistent across both conditions (Supplementary Figure S1). Moreover, the pigmentation in the treated embryos was gradually restored after washing and incubating them in embryo medium without the extract.

### 2.3. GC-MS Analysis of A. valgaris

GC-MS analysis revealed a total of 99 distinct compounds across all four extracts, with each individual extract containing an average of 20–27 identified compounds. The compounds were classified into eleven major chemical categories based on their structural features and functional groups: fatty acids, fatty acid esters, phenolic compounds, terpenoids, glycerides and derivatives, alcohols, hydrocarbons, ketones, steroids, phthalates, amino compounds, and other miscellaneous compounds.

#### 2.3.1. Phytochemical Composition of Ethyl Acetate Extract of *A. vulgaris*

Ethyl acetate extract of *A. vulgaris* yielded 27 compounds with fatty acids (38.8%) and phenolic compounds (26.6%) being the predominant classes (Table 2). The major constituents were 9,12-octadecadienoic acid (linoleic acid, 29.7%), benzenepropanol, 4-hydroxy-α-methyl-, (R)- (26.6%), and 9-octadecenoic acid methyl ester (10.5%). Fatty acid esters contributed 21.7% of the total composition.

#### 2.3.2. Chloroform Extract of *A. vulgaris* Phytochemical Composition

Table 3 presents the phytochemical profile of chloroform extract of *A. vulgaris* which displayed a distinctive phytochemical profile dominated by phenolic compounds (83.3%). The three most abundant compounds were benzenepropanol, 4-hydroxy-α-methyl-, (R)- (46.1%), phenol, p-(2-methylallyl)- (30.5%), and benzaldehyde, 4-hydroxy- (5.9%). This extract also contained notable amounts of ketones (3.5%) and phthalates (3.6%).

#### 2.3.3. Methanol Extract of *A. vulgaris* Phytochemical Composition

The phytochemical composition of the methanol extract of *A. vulgaris* is shown in Table 4 which contains 25 compounds with a balanced distribution between fatty acids (33.5%) and glycerides/derivatives (32.4%). The predominant compounds included 3-O-methyl-d-glucose (29.1%), 9,12-octadecadienoic acid (28.8%), and benzenepropanol, 4-hydroxy-α-methyl-, (R)- (18.8%). Fatty acid esters accounted for 9.4% of the composition.

#### 2.3.4. n-Hexane Extract of *A. vulgaris*—Phytochemical Composition

The phytochemical contents identified in n-hexane extract of *A. vulgaris* are shown in Table 5. n-hexane extract yielded 20 compounds predominantly composed of fatty acid esters (49.6%) and terpenoids (30.0%). The major constituents were 9-octadecenoic acid (Z)-, 2,3-dihydroxypropyl ester (40.3%) and 1,5,5-trimethyl-6-methylene-cyclohexene (21.6%). This extract exhibited the highest concentration of terpenoid compounds among all four extracts.

#### 2.3.5. Comparative Chemical Class Distribution

Table 6 presents a comparative analysis of chemical class distribution across all four extracts. The data reveal extract-specific variations in phytochemical composition that may correspond to differences in solvent polarity and extraction efficiency.

## 3. Discussion

The search for a safe, effective depigmentation agent in zebrafish research is a constant challenge in developmental biology, toxicology, and drug discovery research. This study demonstrates that ethyl acetate extract of *A. vulgaris* effectively inhibits melanogenesis at concentrations as low as 10 µg/mL, with no observed mortality or teratogenic effects throughout 96 h.

PTU, applied at the standard recommended concentration of 30 µg/mL (0.003%), produced comparable depigmentation; however, it is a well-characterized thyroid peroxidase inhibitor and endocrine disruptor. At concentrations overlapping with its effective depigmentation range, PTU has been reported to cause hypothyroid-like phenotypes, craniofacial defects, reduced eye size, and impaired thyroid hormone synthesis in zebrafish embryos [4,5]. It also poses documented occupational health risks to researchers, particularly those of childbearing age, through dermal absorption or accidental ingestion [2,3,7]. PTU did not induce acute mortality at 30 µg/mL. However, at higher concentrations (100 µg/mL, PTU clearly induces the deformities in treated embryos without a significant level of mortalities (Supplementary Figure S7). Of the treated embryos, 100% failed to hatch and exhibited curved trunk and short bodies as compared to ethyl acetate extract of *A. vulgaris*-treated embryos at the same concentration. Most importantly, the endocrine disruption represents a meaningful safety concern that extends beyond acute lethality. When evaluating the toxicity of any depigmentation agent, the concentration at which it exerts its biological effect should be considered, rather than its absolute lethal concentration alone.

Kojic acid required 200 µg/mL to achieve effective depigmentation, a concentration at which it induced cardiac edema and developmental delay in zebrafish embryos, and at which its 24 h LC_50_ (~217 µg/mL) was approached (Table 1). This narrow margin between the effective depigmentation dose and the toxic dose renders kojic acid a less suitable agent for routine laboratory use, in addition to its known carcinogenic potential and limited skin penetration in vivo [9,10].

The ethyl acetate extract of *A. vulgaris* demonstrated a substantially more favorable therapeutic index. Effective depigmentation was achieved at 10–20 µg/mL—a concentration 10-fold lower than that required for kojic acid and modestly lower than that of PTU with no associated mortality, teratogenicity, or developmental abnormality at effective doses. The LC_50_ of the ethyl acetate extract exceeded 1000 µg/mL at 24 h, and no mortality was recorded at 96 h (Table 1), yielding a wide margin between the effective depigmentation concentration and any threshold of observable toxicity. Taken together, these data support the conclusion that the ethyl acetate extract of *A. vulgaris* possesses a superior safety profile relative to both PTU and kojic acid when assessed at their respective effective depigmentation concentrations, which represents the clinically and experimentally relevant basis for comparison.

*A. vulgaris* presents a favorable and competitive safety profile among other botanical depigmenting agents, primarily due to absence of documented cytotoxicity, contact sensitization, or phototoxicity. This safety profile is further supported by its long history of use in traditional medicine and cosmetic applications for skin conditions, with no major adverse effects reported [16]. To date, safety data for *A. vulgaris* in zebrafish is lacking; the direct safety assessment in other animal models is also largely not known. However, the ameliorating effect of *A. vulgaris* extracts in rodents has been documented against liver, kidney, and ovarian damage induced by other toxins [17].

Numerous plant-derived compounds that influence melanogenesis have photodynamic or phototoxic characteristics. By literature review search, we have not found any study indicating phototoxicity of *A. vulgaris*. Studies on *A. vulgaris* and its close relative *Alchemilla mollis* have demonstrated that they do not have phototoxic potential. They are used in skin formulations for safe skin whitening [12,18]. *A. vulgaris* contains high amounts of phenolic compounds, including tannins, flavonoids, and phenolic acids such as gallic and ellagic acids. These compounds inhibit tyrosinase, an enzyme responsible for melanin synthesis, leading to skin whitening without damaging melanocytes [16].

To determine whether the depigmentation observed in zebrafish embryos was due to phototoxicity or true biological activity of the extract, a controlled experiment was conducted. Zebrafish embryos were treated with the extract and subsequently divided into two groups: one incubated overnight under light conditions and the other in complete darkness. In both groups, the extract consistently induced depigmentation regardless of light exposure, suggesting that this effect represents a specific biological response to the extract rather than photobleaching. This conclusion is further supported by the gradual onset of depigmentation observed throughout the incubation period, which contrasts with the rapid depigmentation typically associated with phototoxic agents.

A key finding of this study is the effectiveness of the extract even at 48 h post fertilization (hpf), after melanin synthesis has occurred. This represents a significant advantage over the use of PTU and introduces a novel approach to zebrafish depigmentation methodology. This suggests that the mechanism of action of the extract may involve inhibiting melanosome maturation or promoting melanin degradation, rather than solely blocking initial tyrosinase activity. This characteristic aligns with the cosmetic application of *A. vulgaris* as a skin-whitening agent, given that plant extract products are applied to the skin post-melanin formation to effectively reduce melanin and achieve lighter skin.

The distinct chemical profiles of different extracts provide insight into their biological activities. The chloroform extract, dominated by phenolic compounds (83.3%), exhibited depigmentation activity but significant toxicity, suggesting that high concentrations of certain phenolics may contribute to adverse effects. In contrast, the ethyl acetate extract achieved comparable depigmentation without toxicity. Its unique profile—characterized by a balance of fatty acids (38.8%), phenolic compounds (26.6%), and fatty acid esters (21.7%)—suggests that the combination of these constituents, rather than a single compound, may confer efficacy with a favorable safety margin. The n-hexane and methanol extracts, which lacked significant depigmentation activity, were rich in terpenoids and glycerides respectively, indicating that fatty acids and specific phenolic compounds may be critical for melanin inhibition. *A. vulgaris* ethyl acetate extract is abundant in 9,12-octadecadienoic acid (linoleic acid), which has been documented to inhibit melanogenesis in mammalian melanocytes by affecting tyrosinase activity and promoting melanosome degradation [19].

Several commercially available skin-whitening products are derived from natural sources, for example: (i) kojic acid (extracted from *Aspergillus* spp.), (ii) *Glycyrrhiza glabra* (licorice), (iii) *Morus alba* (mulberry), and (iv) *Curcuma longa* (turmeric). A comprehensive literature review was conducted to systematically compare *A. vulgaris* with these established natural skin-whitening agents across four critical parameters: efficacy, safety, availability, and cost-effectiveness. Preliminary findings, which will be published in a separate report, indicate that *A. vulgaris* offers significant scientific and commercial advantages over the aforementioned natural agents. Nonetheless, a considerable knowledge gap remains; unlike these well-characterized agents, *A. vulgaris* currently lacks mechanistic studies and clinical trial data to substantiate its depigmentation efficacy. To effectively translate the safety and efficacy results of this study into practical clinical applications, future research should focus on well-designed randomized controlled trials, pharmacokinetic characterization, and formulation optimization studies.

### Limitation of the Study

Although many phytoconstituents present in each solvent extract have been identified in this study, the precise compound(s) responsible for depigmentation remain to be isolated and validated through bioassay-guided fractionation. Furthermore, the exact molecular mechanisms underlying direct tyrosinase inhibition, interference with melanocyte development, and other pathways require further elucidation through targeted gene expression and protein analysis. However, the main goal of this study was to propose a safe depigmentation product to be used in zebrafish research as an alternative to PTU. Future studies should assess the potential interference of the extract with the specific biological processes.

## 4. Materials and Methods

### 4.1. Materials

Kojic acid cat # T5889 TargetMol 34 Washington Street, Suite 220, Wellesley Hills, MA, USA. N-Phenylthiourea (PTU) cat# P7629, n-hexane cat # 139386, ethyl acetate cat # 34858, chloroform cat # 650498, and methanol cat # 1.06007 were purchased from Sigma-Aldrich (St. Louis, MO, USA).

### 4.2. Plant Source

*A. vulgaris* (Supplementary Figure S2) was purchased from a local herbal store in Riyadh, Saudi Arabia. A voucher specimen (AV-2024-001) has been deposited at the Department of Zoology Herbarium, King Saud University, Riyadh, Saudi Arabia.

### 4.3. Plant Extraction

Plant material (60 g) was sequentially extracted with n-hexane, chloroform, ethyl acetate, and methanol using the Soxhlet extraction method from least polar to most polar [20]. Solvents were evaporated using a rotary evaporator under vacuum at 40 °C. Extracts were dissolved in methanol to obtain stock solutions. Working solutions were prepared by diluting stock solutions directly into embryo medium (5 mM NaCl, 0.17 mM KCl, 0.33 mM CaCl_2_, 0.33 mM MgSO_4_, 0.5 mg/L methylene blue).

### 4.4. Zebrafish Treatment

#### 4.4.1. Ethical Consideration/IRB Approval

The zebrafish embryos/larvae used in this study were less than five days post fertilization. At this stage, these embryos are typically exempt from regulation by the Institutional Animal Care and Use Committee because they are not considered protected live vertebrate animals until they begin independent feeding [21].

#### 4.4.2. Plant Extract Treatment

Zebrafish maintenance and embryo treatment were performed as previously described [22]. Acute toxicity of *A. vulgaris* in zebrafish embryos was determined according to Organization for Economic Co-operation and Development (OECD) guidelines [23]. Zebrafish embryos were exposed to serial dilutions of plant extracts, PTU, and Kojic acid at two stages: the pre-melanogenesis stage (before the 20-somite stage) and post-melanogenesis (48 h post fertilization hpf). Approximately 30–35 embryos were transferred to 35 mL sterile glass Petri dishes. Embryos were exposed to serial dilutions (0.5, 2.5, 12.5, 62.5, 300, and 1500 µg/mL) of plant extracts in each dish. The 1% methanol (*v*/*v*) was used as a solvent control, and embryo medium as the negative control. The experiments were repeated at least 3 times using different batches of embryos (biological replicates).

To assess whether the observed depigmentation effect was due to the biological activity of the extract rather than photobleaching, batches of embryos were treated with plant extracts and subsequently, each batch was incubated separately in both dark and light conditions at 28 °C.

### 4.5. Melanin Content Determination in Zebrafish

Melanin content was measured following the spectrophotometric method described previously [24] with some modifications. Briefly, pools of 10 embryos per treatment group were homogenized in 1 M NaOH and incubated at 60 °C for 1 h. Absorbance was measured at 405 nm using a synthetic melanin standard curve.

### 4.6. Gas Chromatography Spectrophotometry (GC-MS) Analysis

A total of 1.5 µL (1 mg) sample was injected via an autosampler injection system of gas chromatography–mass spectrometry (GC-MS) 7890B GC system from Agilent Technologies (Santa Clara, CA, USA). The products were identified using database-integrated software (NIST MS vB.10.00). Sample components were analyzed using GC-MS. To separate target compounds, a DB-5 MS capillary column from Agilent technologies (30 m length, 0.25 mm internal diameter, 0.25 μm phase thickness) was used with helium as the carrier gas at a flow rate of 1 mL/min. The inlet temperature was set to 250 °C with a split mode ratio and the oven temperature ranged from 50 to 250 °C, with a total analysis time of 61 min. The sample was held at 50 °C for 1 min, then heated at 4 °C min^−1^ to 250 °C and maintained for 10 min. The MS detector was set as follows: acquisition scan type, mass ranging from 40 to 500 g/mol, scan speed 1.56, 8 min solvent delay, and 230 °C MS source temperature.

### 4.7. Statistical Analysis

Data are presented as mean ± standard deviation of three independent experiments. Statistical significance between the two groups was calculated by one-way ANOVA using SPSS Statistics for Windows (version 17.0; SPSS Inc., Chicago, IL, USA). Statistical significance was set to *p*-value < 0.05. LC50 (µg/mL) values were calculated by probit analysis.

## 5. Conclusions

This study demonstrates that ethyl acetate extract of *A. vulgaris* represents a promising natural alternative to PTU and kojic acid for zebrafish embryo depigmentation. The extract exhibits high efficacy at low concentrations while maintaining a favorable toxicity profile in this model system. Its ability to reduce pigmentation even when administered after melanin synthesis offers potential advantages for experimental designs requiring post-development optical clarity. The use of four different solvents for extraction, combined with sequential toxicity screening, proved effective for obtaining desired biological activity while eliminating harmful constituents. Further investigation of active compounds and mechanisms of action will help establish its role as a valuable tool in zebrafish research.

## Figures and Tables

**Table 1:** Comparative toxicity and teratogenic profiling of different extracts of *A. valgaris*, PTU, and Kojic acid tested in eggs and larvae of zebrafish (*Danio rerio*).

Chemical	n	24 h LC_50_ µg/mL	48 h LC_50_ µg/mL	96 h LC_50_ µg/mL	Cumulative Teratogenicity from All Stages
*A. valgaris* n Hex	60	>1000	0% mortality	ND No mortality	DD 9/60 (*p* = 0.13) DH 19/60 (*p* = 0.0007)
*A. valgaris* CHCl_3_	60	>1000	127.18 ± 1.05 *	ND 100% mortality	DD 18/60 (*p* = 2.77557 × 10^−8^) DH 18/60 (*p* = 2.77557 × 10^−8^) PE 8/60 (*p* = 0.0003)
*A. valgaris* EtOAc	60	>1000	0% mortality	ND No mortality	ND
*A. valgaris* MeOH	60	902 ± 1.05	62.5 ± 1.75	ND No mortality	ND
PTU	60	ND No mortality	ND No mortality	ND No mortality	ND
Kojic acid	60	217.18 ± 1.67	ND No mortality	ND No mortality	CB (*p* = 0.006107081) PE (*p* = 0.0000250579)

* Values are mean of triplicate experiment ± standard deviation. Zebrafish embryos were treated with serial dilutions 1.5, 3.00, 9.00, 30.00, 100.00 and 300 µg/m of each extract and chemical to determine LC_50_. CB: curved bodies; PE: pericardia edema; DD: developmental delay; DH: delayed hatching; ND: not determined/not detected.

**Table 2:** Phytochemical constituents identified in ethyl acetate extract of *A. vulgaris* by GC-MS analysis.

Peak	RT (min)	Area%	Compound Name	Molecular Formula	MW	Chemical Class
1	28.12	26.59	Benzenepropanol, 4-hydroxy-α-methyl-, (R)-	C_10_H_14_O_2_	166	Phenolic Compounds
2	31.25	0.49	1,3-Cyclohexadiene-1-methanol, α,2,6,6-tetramethyl-, (.+-.)-	C_11_H_18_O	166	Terpenoids
3	33.08	1.02	Ethanol, 2-(3,3-dimethylbicyclo[2.2.1]hept-2-ylidene)-	C_11_H_18_O	166	Terpenoids
4	34.18	0.33	Dodecanoic acid, 3-hydroxy-	C_12_H_24_O_3_	216	Other Compounds
5	35.77	0.25	7-Hexadecenoic acid, methyl ester, (Z)-	C_17_H_32_O_2_	268	Fatty Acid Esters
6	35.88	0.59	(Z)-Methyl hexadec-11-enoate	C_17_H_32_O_2_	268	Fatty Acid Esters
7	36.46	3.79	Hexadecanoic acid, methyl ester	C_17_H_34_O_2_	270	Fatty Acid Esters
8	37.12	0.30	Hexadecanoic acid, methyl ester	C_17_H_34_O_2_	270	Fatty Acid Esters
9	37.63	2.53	n-Hexadecanoic acid	C_16_H_32_O_2_	256	Fatty Acids
10	38.14	1.52	12-Methyl-E,E-2,13-octadecadien-1-ol	C_19_H_36_O	280	Alcohols
11	38.35	0.30	Estra-1,3,5(10)-trien-17β-ol	C_18_H_24_O	256	Steroids
12	40.41	3.05	Oleic Acid	C_18_H_34_O_2_	282	Fatty Acids
13	40.59	10.53	9-Octadecenoic acid (Z)-, methyl ester	C_19_H_36_O_2_	296	Fatty Acid Esters
14	40.75	0.90	11-Octadecenoic acid, methyl ester	C_19_H_36_O_2_	296	Fatty Acid Esters
15	41.23	1.23	Methyl stearate	C_19_H_38_O_2_	298	Fatty Acid Esters
16	41.80	29.74	9,12-Octadecadienoic acid (Z,Z)-	C_18_H_32_O_2_	280	Fatty Acids
17	42.62	0.91	9,12-Octadecadienoic acid (Z,Z)-, methyl ester	C_19_H_34_O_2_	294	Fatty Acid Esters
18	42.77	0.80	9-Octadecenoic acid, (E)-	C_18_H_34_O_2_	282	Fatty Acids
19	43.97	0.41	cis-13-Eicosenoic acid	C_20_H_38_O_2_	310	Fatty Acids
20	44.92	1.60	9,12-Octadecadienoic acid (Z,Z)-, 2-hydroxy-1-(hydroxymethyl)ethyl ester	C_21_H_38_O_4_	354	Other Compounds
21	47.02	1.08	cis-13,16-Docasadienoic acid	C_22_H_40_O_2_	336	Fatty Acids
22	47.48	0.81	E)-13-Docosenoic acid	C_22_H_42_O_2_	338	Fatty Acids
23	47.61	2.43	9-Octadecenoic acid (Z)-, 2,3-dihydroxypropyl ester	C_21_H_40_O_4_	356	Fatty Acids
24	48.41	0.96	Undec-10-ynoic acid, undecyl ester	C_22_H_40_O_2_	336	Fatty Acid Esters
25	48.54	7.08	Glycidyl oleate	C_21_H_38_O_3_	338	Glycerides and Derivatives
26	49.09	0.21	Ethanol, 2-(9-octadecenyloxy)-, (Z)-	C_20_H_40_O_2_	312	Alcohols
27	49.61	0.54	Di-n-octyl phthalate	C_24_H_38_O_4_	390	Phthalates

**Table 3:** Phytochemical constituents identified in Chloroform extract of *A. vulgaris* by GC-MS analysis.

Peak	RT (min)	Area%	Compound Name	Molecular Formula	MW	Chemical Class
1	18.91	0.20	2-Methoxy-4-vinylphenol	C_9_H_10_O_2_	150	Phenolic Compounds
2	22.16	5.88	Benzaldehyde, 4-hydroxy-	C_7_H_6_O_2_	122	Phenolic Compounds
3	25.44	0.08	(1R,3E,7E,11R)-1,5,5,8-Tetramethyl-12-oxabicyclo[9.1.0]dodeca-3,7-diene	C_15_H_24_O	220	Terpenoids
4	27.18	3.49	2-Butanone, 4-(4-hydroxyphenyl)-	C_10_H_12_O_2_	164	Ketones
5	28.61	46.09	Benzenepropanol, 4-hydroxy-α-methyl-, (R)-	C_10_H_14_O_2_	166	Phenolic Compounds
6	29.10	30.51	Phenol, p-(2-methylallyl)-	C_10_H_12_O	148	Phenolic Compounds
7	29.87	0.20	Benzenepropanol, 4-hydroxy-3-methoxy-	C_10_H_14_O_3_	182	Phenolic Compounds
8	30.18	0.28	4-(3-Aminobutyl)-2-methoxyphenol	C_11_H_17_NO_2_	195	Phenolic Compounds
9	30.31	0.13	2-Cyclohexen-1-one, 4-(3-hydroxy-1-butenyl)-3,5,5-trimethyl-, [R-[R*,R*-(E)]]-	C_13_H_20_O_2_	208	Other Compounds
10	34.21	0.32	9-Eicosyne	C_20_H_38_	278	Hydrocarbons
11	34.82	0.41	E,E-6,8-Tridecadien-2-ol, acetate	C_15_H_26_O_2_	238	Other Compounds
12	35.30	0.07	γ-Linolenic acid, methyl ester	C_19_H_32_O_2_	292	Fatty Acid Esters
13	36.03	0.91	1,2-Benzenedicarboxylic acid, butyl 2-methylpropyl ester	C_16_H_22_O_4_	278	Other Compounds
14	36.45	0.53	Hexadecanoic acid, methyl ester	C_17_H_34_O_2_	270	Fatty Acid Esters
15	37.20	0.72	5-Benzofuranacetic acid, 6-ethenyl-2,4,5,6,7,7a-hexahydro-3,6-dimethyl-α-methylene-2-oxo-, methyl	C_16_H_20_O_4_	276	Fatty Acid Esters
16	37.62	0.38	Pentadecanoic acid	C_15_H_30_O_2_	242	Fatty Acids
17	37.85	0.58	n-Hexadecanoic acid	C_16_H_32_O_2_	256	Fatty Acids
18	38.32	0.10	12-Methyl-E,E-2,13-octadecadien-1-ol	C_19_H_36_O	280	Alcohols
19	38.45	0.07	Estra-1,3,5(10)-trien-17β-ol	C_18_H_24_O	256	Steroids
20	39.47	0.16	2,4,7,14-Tetramethyl-4-vinyl-tricyclo[5.4.3.0(1,8)]tetradecan-6-ol	C_20_H_34_O	290	Alcohols
21	40.35	0.42	Oleic Acid	C_18_H_34_O_2_	282	Fatty Acids
22	40.45	0.05	9-Octadecenoic acid (Z)-, methyl ester	C_19_H_36_O_2_	296	Fatty Acid Esters
23	40.64	0.52	11-Octadecenoic acid, methyl ester	C_19_H_36_O_2_	296	Fatty Acid Esters
24	40.88	0.44	Methyl stearate	C_19_H_38_O_2_	298	Fatty Acid Esters
25	42.06	3.31	9,12-Octadecadienoic acid (Z,Z)-, methyl ester	C_19_H_34_O_2_	294	Fatty Acid Esters
26	49.73	3.63	Di-n-octyl phthalate	C_24_H_38_O_4_	390	Phthalates
27	56.86	0.52	Ethyl iso-allocholate	C_26_H_44_O_5_	436	Phenolic Compounds

**Table 4:** Phytochemical constituents identified in methanol extract of *A. vulgaris* by GC-MS analysis.

Peak	RT (min)	Area%	Compound Name	Molecular Formula	MW	Chemical Class
1	23.71	0.31	Ethyl 2-[(4-methylphenyl)amino]propanoate	C_12_H_17_NO_2_	207	Amino Compounds
2	27.95	18.83	Benzenepropanol, 4-hydroxy-α-methyl-, (R)-	C_10_H_14_O_2_	166	Phenolic Compounds
3	34.62	29.11	3-O-Methyl-d-glucose	C_7_H_14_O_6_	194	Glycerides and Derivatives
4	36.46	1.20	Pentadecanoic acid, 14-methyl-, methyl ester	C_17_H_34_O_2_	270	Fatty Acid Esters
5	37.11	0.48	Hexadecanoic acid, methyl ester	C_17_H_34_O_2_	270	Fatty Acid Esters
6	37.64	3.44	n-Hexadecanoic acid	C_16_H_32_O_2_	256	Fatty Acids
7	38.14	0.10	12-Methyl-E,E-2,13-octadecadien-1-ol	C_19_H_36_O	280	Alcohols
8	38.35	0.13	Estra-1,3,5(10)-trien-17β-ol	C_18_H_24_O	256	Steroids
9	40.08	0.18	9,12-Octadecadienoyl chloride, (Z,Z)-	C_18_H_31_ClO	298	Other Compounds
10	40.41	1.00	Oleic Acid	C_18_H_34_O_2_	282	Fatty Acids
11	40.59	3.74	9-Octadecenoic acid (Z)-, methyl ester	C_19_H_36_O_2_	296	Fatty Acid Esters
12	40.75	0.28	11-Octadecenoic acid, methyl ester	C_19_H_36_O_2_	296	Fatty Acid Esters
13	41.23	0.21	Methyl stearate	C_19_H_38_O_2_	298	Fatty Acid Esters
14	41.88	28.83	9,12-Octadecadienoic acid (Z,Z)-	C_18_H_32_O_2_	280	Fatty Acids
15	42.58	0.39	9,12-Octadecadienoic acid (Z,Z)-, methyl ester	C_19_H_34_O_2_	294	Fatty Acid Esters
16	43.97	0.22	cis-13-Eicosenoic acid	C_20_H_38_O_2_	310	Fatty Acid Esters
17	44.92	0.67	9,12-Octadecadienoic acid (Z,Z)-, 2-hydroxy-1-(hydroxymethyl)ethyl ester	C_21_H_38_O_4_	354	Other Compounds
18	46.37	0.54	Undec-10-ynoic acid, dodecyl ester	C_23_H_42_O_2_	350	Fatty Acid Esters
19	47.47	0.48	Z-(13,14-Epoxy)tetradec-11-en-1-ol acetate	C_16_H_28_O_3_	268	Other Compounds
20	47.61	1.31	9-Octadecenoic acid (Z)-, 2,3-dihydroxypropyl ester	C_21_H_40_O_4_	356	Glycerides and Derivatives
21	48.41	0.79	Undec-10-ynoic acid, undecyl ester	C_22_H_40_O_2_	336	Fatty Acid Esters
22	48.54	3.33	Glycidyl oleate	C_21_H_38_O_3_	338	Other Compounds
23	48.80	0.18	n-Propyl 11-octadecenoate	C_21_H_40_O_2_	324	Alcohols
24	49.08	0.18	Ethanol, 2-(9-octadecenyloxy)-, (Z)-	C_20_H_40_O_2_	312	Fatty Acid Esters
25	53.07	4.07	Cholestan-3-ol, 2-methylene-, (3β,5α)-	C_28_H_48_O	400	Terpenoids

**Table 5:** Phytochemical constituents identified in n-hexane extract by GC-MS analysis.

Peak	RT (min)	Area%	Compound Name	Molecular Formula	MW	Chemical Class
1	17.46	1.08	Bicyclo[3.2.2]non-6-en-3-one	C_9_H_12_O	136	Terpenoids
2	18.46	0.64	Allyl o-tolyl ether	C_10_H_12_O	148	Other Compounds
3	20.22	21.57	1,5,5-Trimethyl-6-methylene-cyclohexene	C_10_H_16_	136	Terpenoids
4	27.31	0.60	Bicyclo[3.1.1]hept-2-ene-2-ethanol, 6,6-dimethyl-	C_11_H_18_O	166	Terpenoids
5	34.22	2.54	9-Eicosyne	C_20_H_38_	278	Hydrocarbons
6	34.37	1.37	7,11-Hexadecadienal	C_16_H_28_O	236	Other Compounds
7	34.85	0.77	E,E-6,8-Tridecadien-2-ol, acetate	C_15_H_26_O_2_	238	Other Compounds
8	35.31	1.04	γ-Linolenic acid, methyl ester	C_19_H_32_O_2_	292	Fatty Acid Esters
9	36.52	0.70	Pentadecanoic acid, 14-methyl-, methyl ester	C_17_H_34_O_2_	270	Fatty Acid Esters
10	40.65	2.21	9-Octadecenoic acid (Z)-, methyl ester	C_19_H_36_O_2_	296	Fatty Acid Esters
11	40.90	1.85	11-Octadecenoic acid, methyl ester	C_19_H_36_O_2_	296	Fatty Acid Esters
12	41.40	3.10	Methyl stearate	C_19_H_38_O_2_	298	Fatty Acid Esters
13	45.57	2.37	Z-(13,14-Epoxy)tetradec-11-en-1-ol acetate	C_16_H_28_O_3_	268	Other Compounds
14	46.03	4.98	Z,E-3,13-Octadecadien-1-ol	C_18_H_34_O	266	Alcohols
15	47.72	40.28	9-Octadecenoic acid (Z)-, 2,3-dihydroxypropyl ester	C_21_H_40_O_4_	356	Glycerides and Derivatives
16	48.62	3.49	Glycidyl oleate	C_21_H_38_O_3_	338	Alcohols
17	49.68	1.20	Di-n-octyl phthalate	C_24_H_38_O_4_	390	Phthalates
18	52.04	3.56	6-epi-shyobunol	C_15_H_26_O	222	Terpenoids
19	52.70	3.46	Octadecanoic acid, 4-hydroxy-, methyl ester	C_19_H_38_O_3_	314	Fatty Acid Esters
20	53.11	3.17	Cholestan-3-ol, 2-methylene-, (3β,5α)-	C_28_H_48_O	400	Terpenoids

**Table 6:** Comparative chemical class distribution across extracts.

Chemical Class	n-Hexane	Ethyl Acetate	Chloroform	Methanol
Alcohols	2 (5.8%)	2 (1.7%)	-	-
Fatty Acid Esters	6 (49.5%)	9 (21.7%)	6 (5.4%)	9 (9.4%)
Fatty Acids	-	8 (38.8%)	4 (2.1%)	4 (33.5%)
Glycerides and Derivatives	1 (3.5%)	1 (7.1%)	-	2 (32.4%)
Ketones	-	-	1 (3.5%)	-
Other Compounds	4 (7.5%)	2 (1.8%)	-	-
Phenolic Compounds	-	1 (26.6%)	7 (83.3%)	1 (18.8%)
Phthalates	-	-	1 (3.6%)	-
Terpenoids	5 (30.0%)	2 (1.5%)	-	1 (4.1%)

Values represent: Number of compounds (Total Area%).

## Data Availability

The original contributions presented in this study are included in the article/Supplementary Material. Further inquiries can be directed to the corresponding author.

## Supplementary Materials

The following supporting information can be downloaded at https://www.mdpi.com/article/10.3390/ph19050714/s1, Figure S1: zebrafish embryos treated with ethyl acetate extract of *A. vulgaris* and subsequently incubated under light and dark condition for 3 days. Figure S2: Plant purchased from local herbal store. Figure S3: GC-MS chromatogram of ethyl acetate extract. Figure S4: GC-MS chromatogram of CHCL_3_ extract. Figure S5: GC-MS chromatogram of methanol extract. Figure S6: GC-MS chromatogram of nHex extract of *A. vulgaris*. Figure S7: zebrafish embryos treated with 100 µg/mL PTU.
