# Supplementary material for: A Safe Natural Alternative to Phenylthiourea: Ethyl Acetate Extract of Alchemilla vulgaris for Zebrafish Embryo Depigmentation
Source: Pharmaceuticals (Basel). 2026 Apr 30;19(5):714. doi: 10.3390/ph19050714 (PMC13209467; doi:10.3390/ph19050714)

File :C:\Users\user\Desktop\GC M S\2026\M34.D
Operator : 5-AA53-003\Administrator
Acquired : 02 Dec 2025 21:09 using AcqMethod NASSER2024_1.M
Instrument : GCMS
Sample Name: M34
Misc Info :
Vial Number: 1

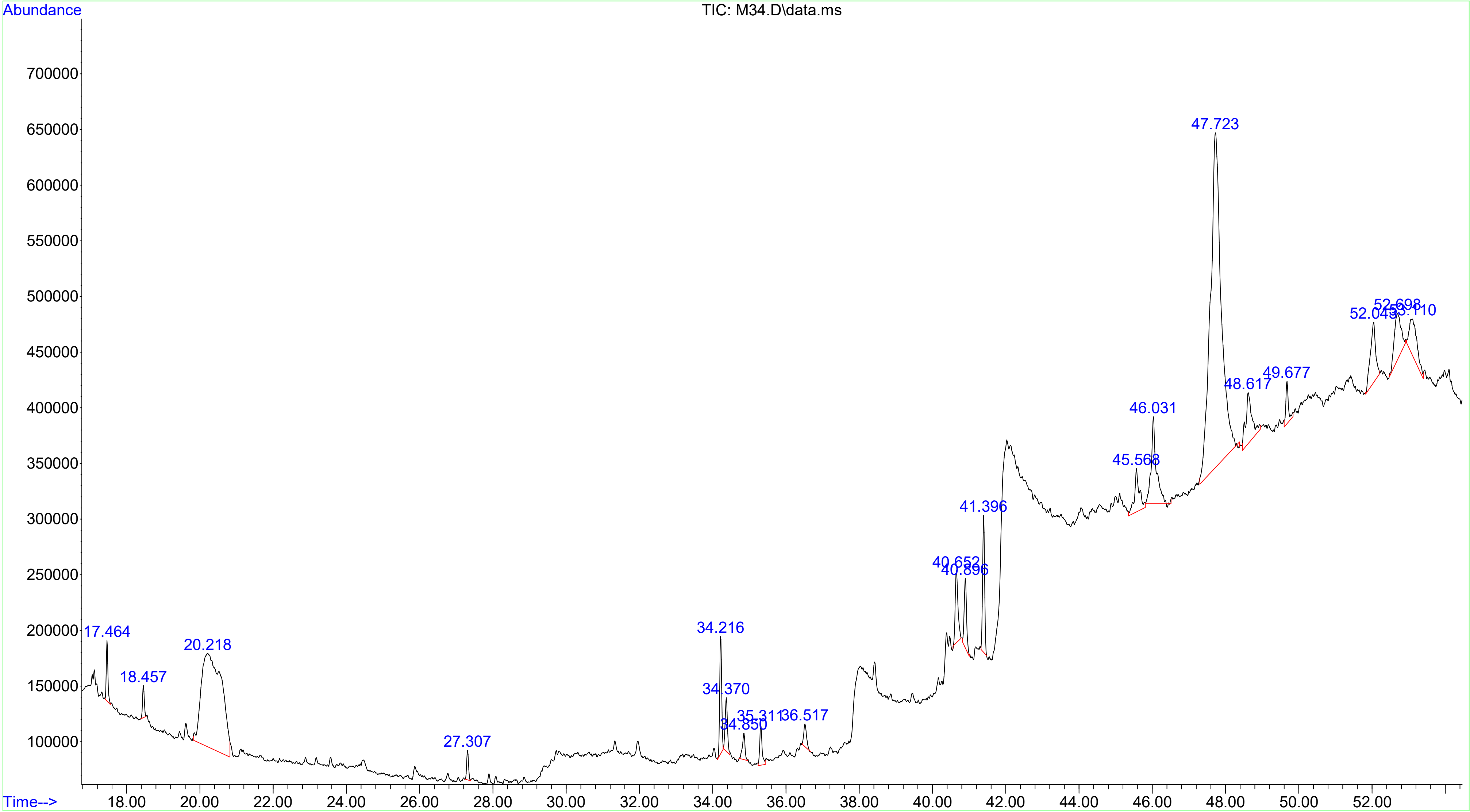

Supplement: Supplementary file 1 [file pharmaceuticals-19-00714-s001.zip › Figure S4B- nHex.pdf]
