# Supplementary figures and images for: A Safe Natural Alternative to Phenylthiourea: Ethyl Acetate Extract of Alchemilla vulgaris for Zebrafish Embryo Depigmentation
Source: Pharmaceuticals (Basel). 2026 Apr 30;19(5):714. doi: 10.3390/ph19050714 (PMC13209467; doi:10.3390/ph19050714)

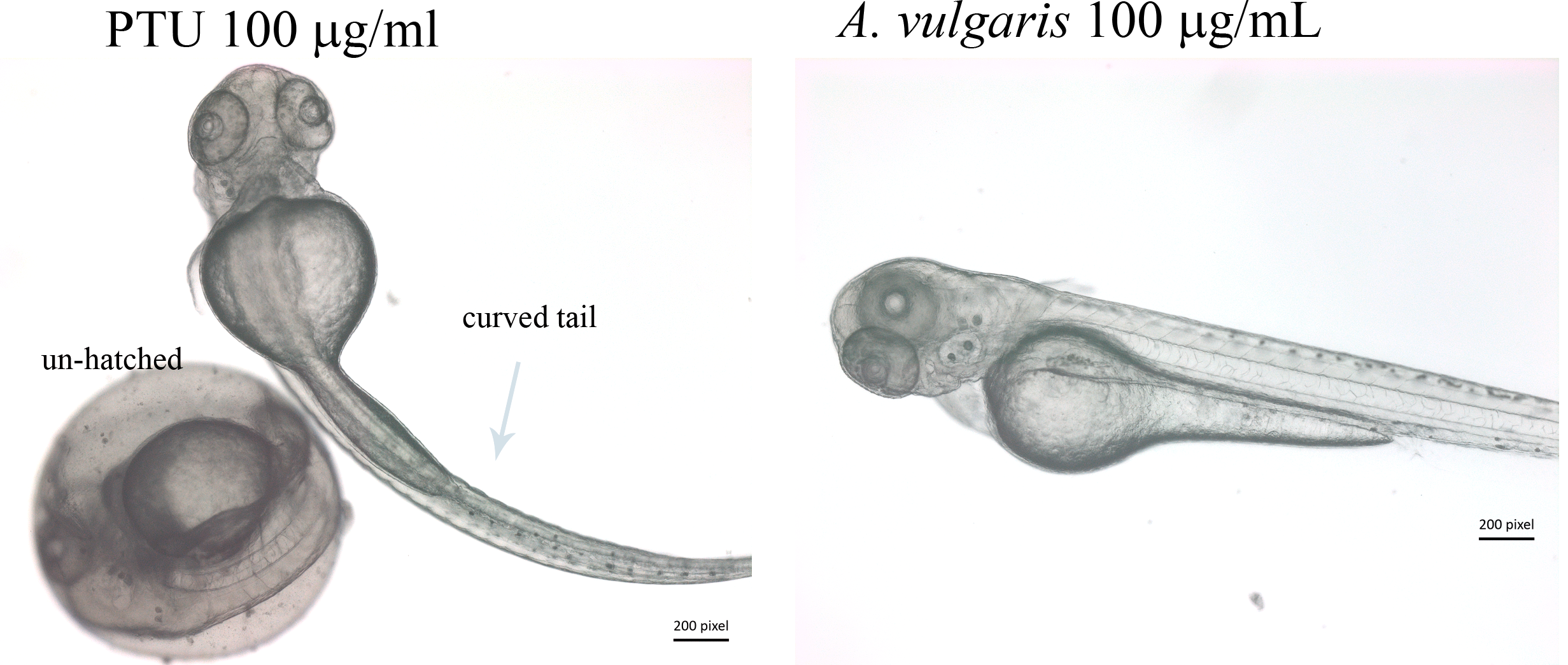

Supplement: Supplementary file 1 [file pharmaceuticals-19-00714-s001.zip › Figure S7.tif]

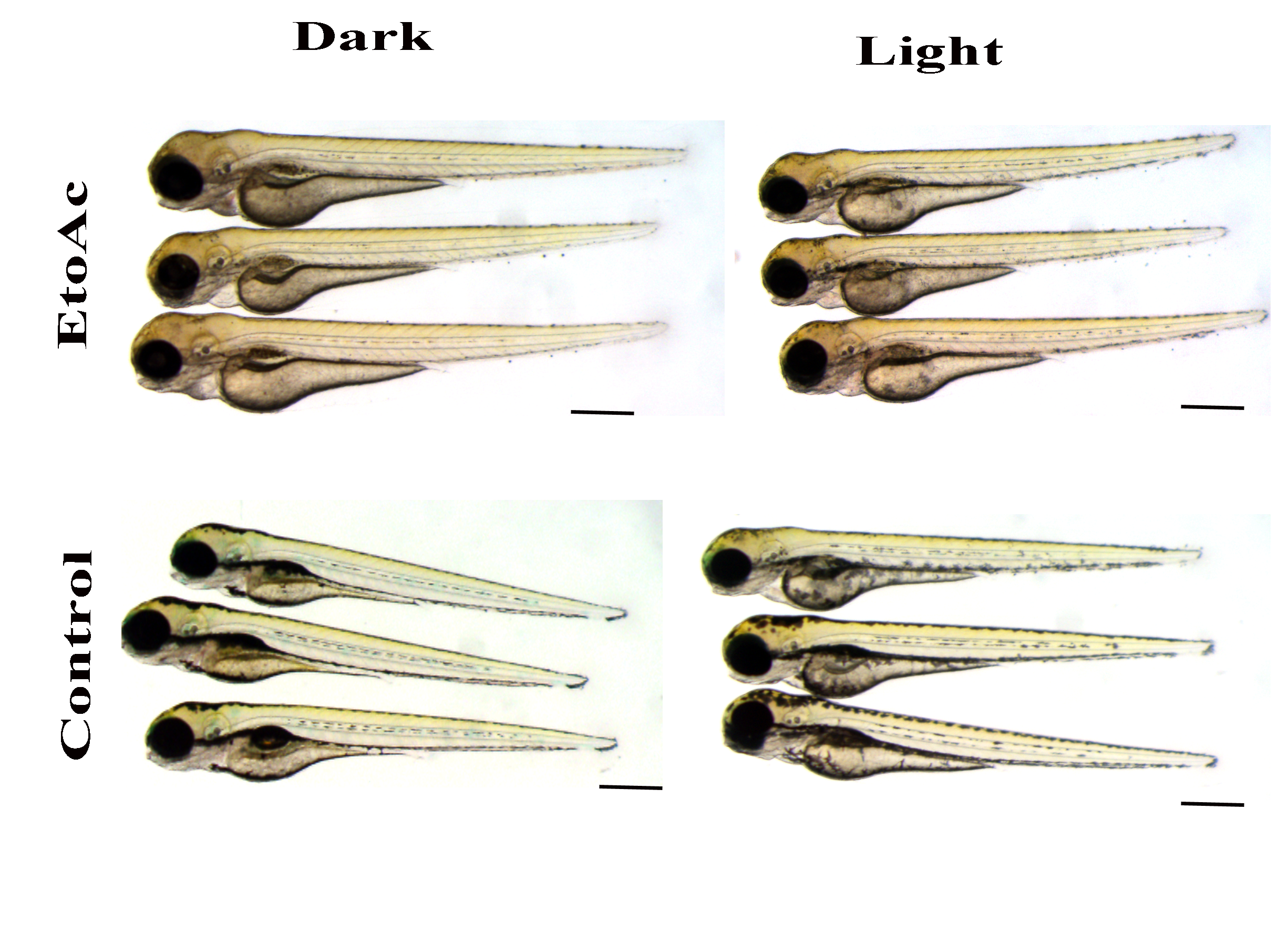

Supplement: Supplementary file 1 [file pharmaceuticals-19-00714-s001.zip › Figure S1.tif]

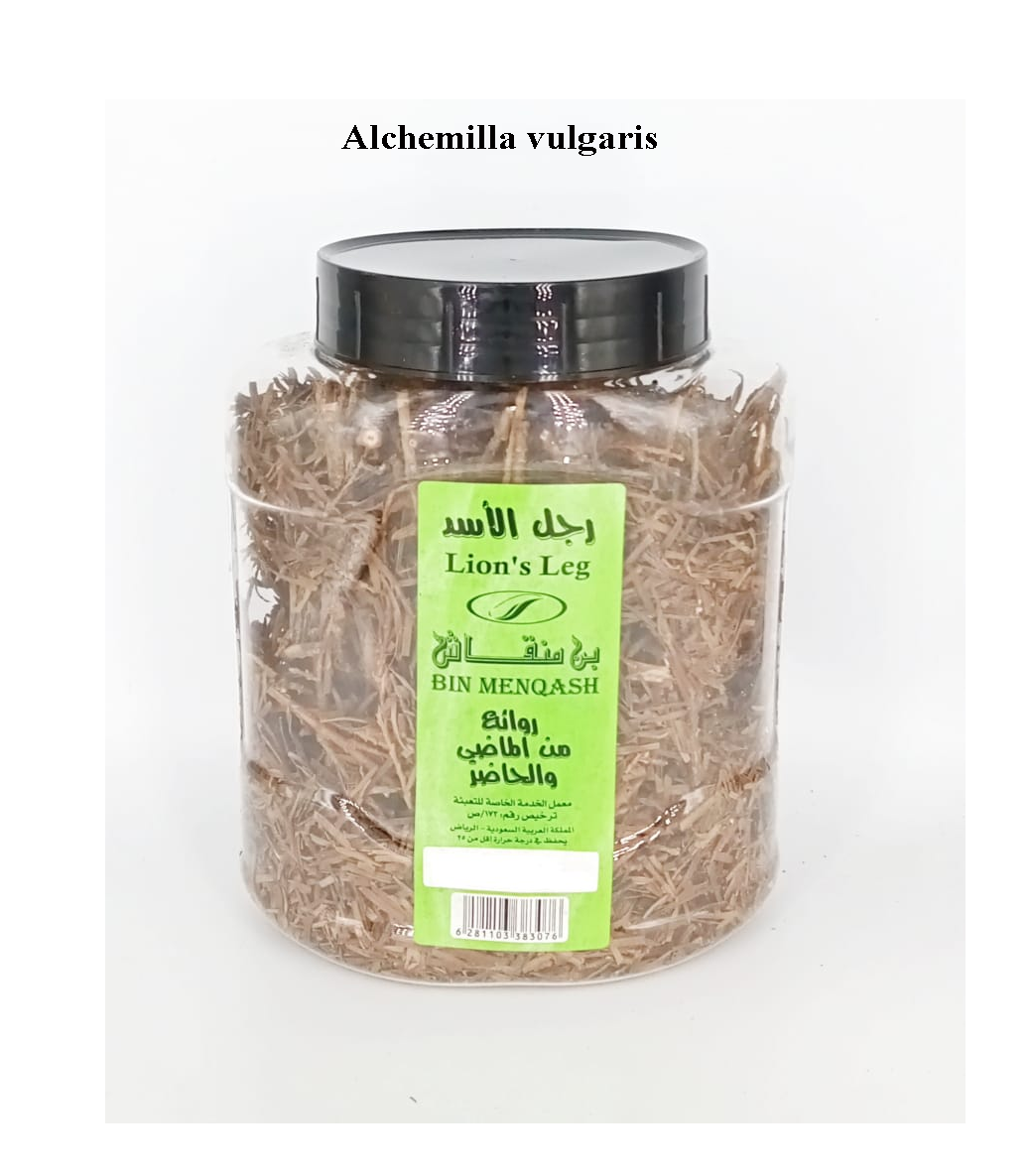

Supplement: Supplementary file 1 [file pharmaceuticals-19-00714-s001.zip › Figure S2B.tif]
